# Supplementary material for: Influence of Sex and Age on Site of Onset, Morphology, and Site of Metastasis in Colorectal Cancer: A Population-Based Study on Data from Four Italian Cancer Registries
Source: Cancers (Basel). 2023 Jan 28;15(3):803. doi: 10.3390/cancers15030803 (PMC9913256; doi:10.3390/cancers15030803)

Table S1. Raggruppamento in gruppi morfologici.

Morphology group	Morphology codes (ICD-O-3 M)
Adenocarcinoma	8140/3, 8144/3, 8201/3, 8210/3, 8211/3, 8220/3, 8230/3, 8260/3, 8261/3, 8262/3, 8263/3, 8310/3, 8323/3, 8440/3, 8470/3, 8510/3, 8560/3, 8574/3
Mucinous carcinoma	8480/3, 8481/3, 8490/3
Other	8013/3, 8020/3, 8021/3, 8051/3, 8070/3, 8071/3, 8072/3, 8123/3, 8240/3, 8241/3, 8243/3, 8245/3, 8246/3, 8249/3, 8720/3, 8800/3, 8801/3, 8804/3, 8850/3, 8890/3, 8936/3, 9140/3, 9680/3, 9687/3
NOS	8000/3, 8001/3, 8010/3

NOS: Not Otherwise Specified

Table S2. Distribuzione assoluta e percentuale delle principali variabili nella coorte di pazienti con tumore al colon.

N = 10808	n (%)
Sex	
Male	5890 (54.5)
Female	4918 (45.5)
Age groups	
<50	485 (4.5)
50-69	3673 (34.0)
>69	6650 (61.5)
Site	
Right colon	3939 (36.4)
Left colon	4095 (37.9)
Rectum	2001 (18.5)
NOS	773 (7.2)
Morphologies	
Adenocarcinoma	8786 (81.3)
Mucinous carcinoma	1089 (10.1)
Other	155 (1.4)
NOS	778 (7.2)
Metastasis	
No	5484 (50.7)
Yes	2038 (18.9)
Unknown	3286 (30.4)
Age (Mean ± SD)	71.8 ± 12.0
Male	70.9 ± 11.4
Female	73.0 ± 12.6

NOS: Not Otherwise Specified

Figure S1. Sopravvivenza relativa dei pazienti metastatici alla diagnosi per classe d'età, sesso e sito di insorgenza del tumore.

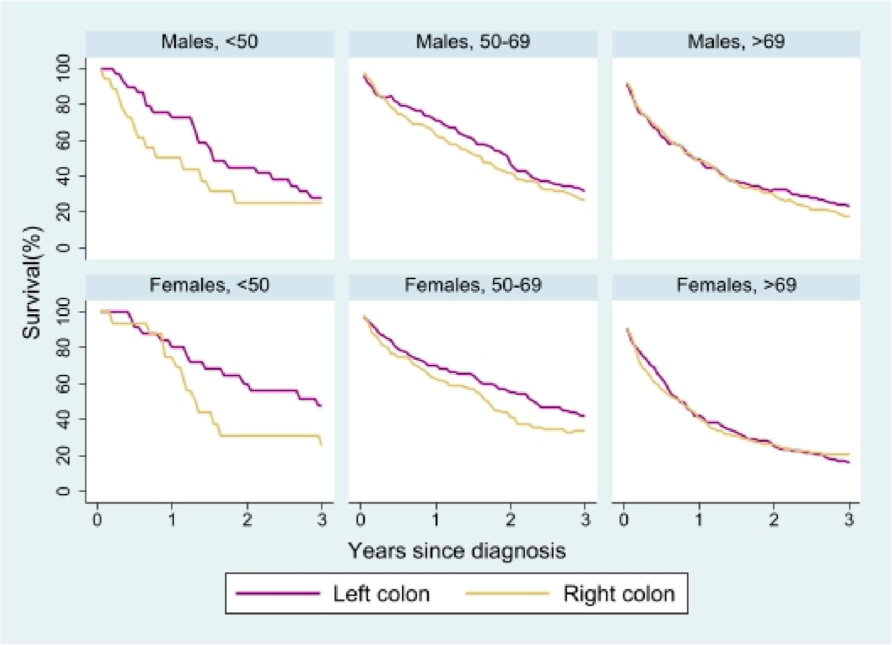

Figure S2. Sopravvivenza relativa dei pazienti metastatici alla diagnosi per classe d'età, sesso e morfologia del tumore.

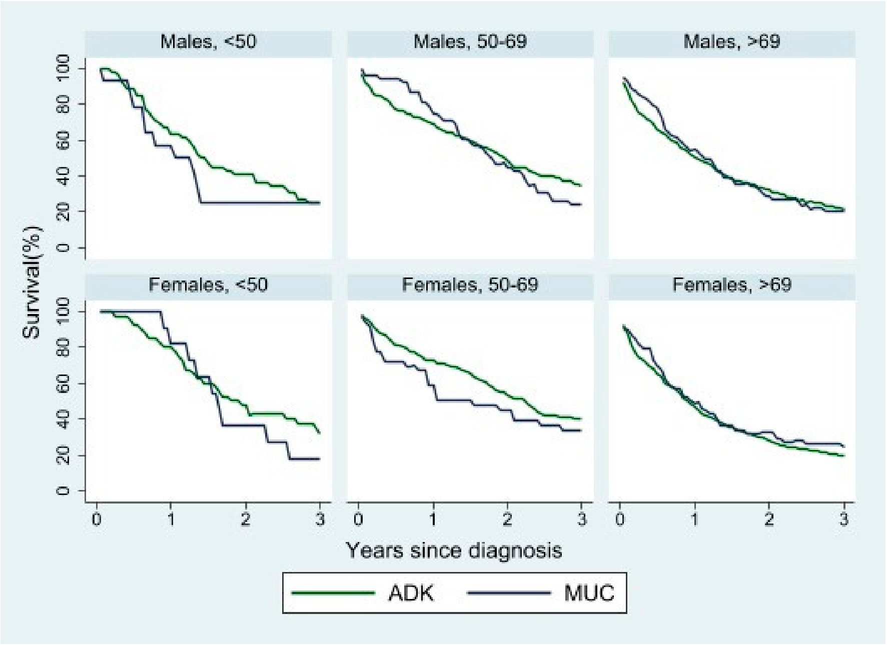

Supplement: Supplementary file 1 [file cancers-15-00803-s001.zip › cancers-2070248-supplementary.pdf]
